# Oncogenic RAC1 and NRAS drive resistance to endoplasmic reticulum stress through MEK/ERK signalling

**DOI:** 10.1016/j.cellsig.2018.01.004

**Published:** 2018-04

**Authors:** Michael D. Bright, Paul A. Clarke, Paul Workman, Faith E. Davies

**Affiliations:** The Institute of Cancer Research, 15 Cotswold Road, Sutton SM2 5NG, UK

**Keywords:** RAC1, NRAS, MEK, ERK, ER-stress

## Abstract

Cancer cells are able to survive under conditions that cause endoplasmic reticulum stress (ER-stress), and can adapt to this stress by upregulating cell-survival signalling pathways and down-regulating apoptotic pathways. The cellular response to ER-stress is controlled by the unfolded protein response (UPR). Small Rho family GTPases are linked to many cell responses including cell growth and apoptosis. In this study, we investigate the function of small GTPases in cell survival under ER-stress. Using siRNA screening we identify that RAC1 promotes cell survival under ER-stress in cells with an oncogenic N92I RAC1 mutation. We uncover a novel connection between the UPR and N92I RAC1, whereby RAC1 attenuates phosphorylation of EIF2S1 under ER-stress and drives over-expression of ATF4 in basal conditions. Interestingly, the UPR connection does not drive resistance to ER-stress, as knockdown of ATF4 did not affect this. We further investigate cancer-associated kinase signalling pathways and show that RAC1 knockdown reduces the activity of AKT and ERK, and using a panel of clinically important kinase inhibitors, we uncover a role for MEK/ERK, but not AKT, in cell viability under ER-stress. A known major activator of ERK phosphorylation in cancer is oncogenic NRAS and we show that knockdown of NRAS in cells, which bear a Q61 NRAS mutation, sensitises to ER-stress. These findings highlight a novel mechanism for resistance to ER-stress through oncogenic activation of MEK/ERK signalling by small GTPases.

## Highlights

• This study uncovers links between oncogenic RAC1 and NRAS, and cell survival under ER-stress.
• N92I Rac1 influences the Unfolded Protein Response, and promotes cell survival under ER-stress through MEK/ERK signalling.
• Inhibition of MEK/ERK signaling, or knockdown of NRAS in NRAS-mutant cell lines causes sensitivity to ER-stress.
• These findings suggest that MEK/ERK signaling through RAC1 and NRAS drives resistance to ER-stress in cancer cells.

## 1 Introduction

Oncogenesis and uncontrolled cancer cell division often lead to conditions that perturb protein folding and induce endoplasmic reticulum stress (ER-stress) [1]. These conditions, such as protein overexpression and hypoxia, can lead to cell death if cancer cells do not adapt. Cell fate under ER-stress is controlled by the unfolded protein response (UPR), a transcriptional programme that promotes increased protein folding capacity and adaptation to ER-stress or, if stress is not resolved, activates apoptotic pathways [2]. There are three ubiquitously expressed controlling arms to the UPR: endoplasmic reticulum to nucleus signalling 1 (ERN1, hereafter referred to by its common name, IRE1); eukaryotic translation initiation factor 2-alpha kinase 3 (EIF2AK3, hereafter referred to by its common name, PERK); and cyclic AMP-dependent transcription factor ATF-6 alpha (ATF6). IRE1 bears a kinase domain and a ribonuclease (RNase) domain, and dimerizes/oligomerizes upon activation by ER-stress. The main target of the IRE1 RNase domain is the mRNA coding for X-box-binding protein 1 (XBP1) [3]. IRE1 cleaves full length *XBP1* mRNA (*XBP1u*) preceding its non-conventional splicing within the cytoplasm. Spliced *XBP1* mRNA (*XBP1s*) codes for the active XBP1 transcription factor which promotes adaptation to stress [4]. PERK is a kinase that dimerizes upon activation by ER-stress [5] and phosphorylates its major target, eukaryotic translation initiation factor 2 subunit alpha (EIF2S1). This phosphorylation event attenuates protein translation relieving the protein folding burden within the ER and promoting cell survival [6]. However, translation of pro-apoptotic transcription factors such as cyclic AMP-dependent transcription factor ATF-4 (ATF4) and DNA damage-inducible transcript 3 (DDIT3) continues, promoting apoptosis if ER-stress is not resolved [7]. ATF6 is a transcription factor that is held within the ER membrane in resting conditions. Upon ER-stress, ATF6 is released from the ER and traffics to the Golgi apparatus where it is cleaved, releasing the active transcription factor to translocate to the nucleus. A major function of ATF6 is to promote adaptation to stress by increasing expression of pro-survival factors [8]. Under acute ER-stress, the UPR promotes cell survival and adaptation. However, this function is superseded by pro-apoptotic signalling pathways (e.g. ATF4 and DDIT3) under prolonged ER-stress [9].

Oncogenic signalling in cancer cells promotes the avoidance of apoptosis through multiple mechanisms [10]. A key family of signalling proteins that has been linked to tumorigenesis and evasion of apoptosis is the Rho GTPases—a family of 20 GTPases, several of which have been found to be overexpressed or mutated in cancer [11]. One of the most well-characterised Rho GTPases is Ras-related C3 botulinum toxin substrate 1 (RAC1). Both overexpression and mutation have been described in cancer, most notably, RAC1 P29S mutation is a recurrent mutation in melanoma [12]. A major role of RAC1 is as a master regulator of cell migration, which is of particular importance to metastasis and angiogenesis in cancer [13]. RAC1 has also been linked to the strongly cancer-associated kinase signalling pathways involving isoforms of phosphatidylinositol-4,5-bisphosphate 3-kinase (PI3K), RAC-alpha serine, threonine protein-kinase (AKT), and serine/threonine protein-kinase mTOR (MTOR) (hereafter referred to as the PI3K/AKT/MTOR pathway) [14], and Raf proto-oncogene (RAF)/mitogen-activated protein kinase kinase (MEK)/Mitogen activated protein kinase (ERK) (hereafter referred to as the RAF/MEK/ERK pathway) [12, 15]. Among many other effects, these pathways are involved in the avoidance of apoptosis and drug resistance [16]. RAC1 signalling has also recently been linked to drug resistance [17] and P29S mutant RAC1 promotes resistance to inhibition of RAF and MEK [18].

The roles of Rho GTPases in ER-stress signalling and the UPR have not yet been fully established. In *Caenorhabditis elegans*, knockout of the Rho GTPase CRP-1 impairs the UPR and leads to stress-sensitivity [19], suggesting that human Rho GTPases may also affect cell survival under ER-stress. The major function of the Rho GTPase family is in control of cell migration and important components of this process are actin and myosin [20]. Myosin IIB has recently been implicated in the oligomerization and clustering of IRE1 [21] suggesting that upstream Rho GTPases may feed into this process. In the present work, we aimed to clarify the role of Rho GTPases in cancer cell survival under ER-stress. Taking an siRNA approach we found that RAC1 knockdown in HT-1080 human fibrosarcoma cells that express N92I oncogenic RAC1 sensitises them to ER-stress. Investigating the UPR in these cells, we found that knockdown of RAC1 reduced the expression of ATF4 mRNA and protein, and also reduced phosphorylation of EIF2S1. However, these effects on UPR signalling were not responsible for the increased sensitivity to ER-stress. Further investigation of kinase signalling pathways showed that RAC1 knockdown impairs the basal phosphorylation of AKT, MEK1/2 and ERK1/2. Using clinically relevant PI3K/AKT/MTOR or RAF/MEK/ERK inhibitors we found that inhibition of MEK/ERK, but not PI3K/AKT/MTOR pathways, caused sensitivity to ER-stress. The B-Raf proto-oncogene, serine/threonine kinase (BRAF) inhibitor GDC-0879 induced paradoxical ERK1/2 activation as previously described [22], and partially rescued the effect of RAC1 knockdown. Because oncogenic NRAS also drives ERK signalling in cancer, we tested the effect of NRAS knockdown in HT-1080 and RD soft tissue sarcoma cell lines (which both contain a Q61 NRAS mutation) and found that NRAS knockdown sensitised cells to ER-stress. We conclude that oncogenic RAC1 and NRAS mutations protect cancer cells from ER-stress by activating ERK1/2.

## 2 Materials and methods

### 2.1 Cell culture and induction of ER-stress

HT-1080 and RD soft tissue sarcoma cell lines were obtained from the European Collection of Cell Cultures and the American Type Culture Collection, respectively. Cells were used within two months of thawing and were authenticated by the suppliers. Cells were maintained at 37 °C, 5% CO_2_, in Minimal Essential Media supplemented with 1.87 mM Glutamax (ThermoFisher Scientific) and 10% heat-inactivated foetal bovine serum (Life Technologies). ER-stress was induced by the addition of 2 mM dithiothreitol (DTT) or 20 μg/ml tunicamycin (Tm).

### 2.2 siRNA transfection

**Fig. 1:**
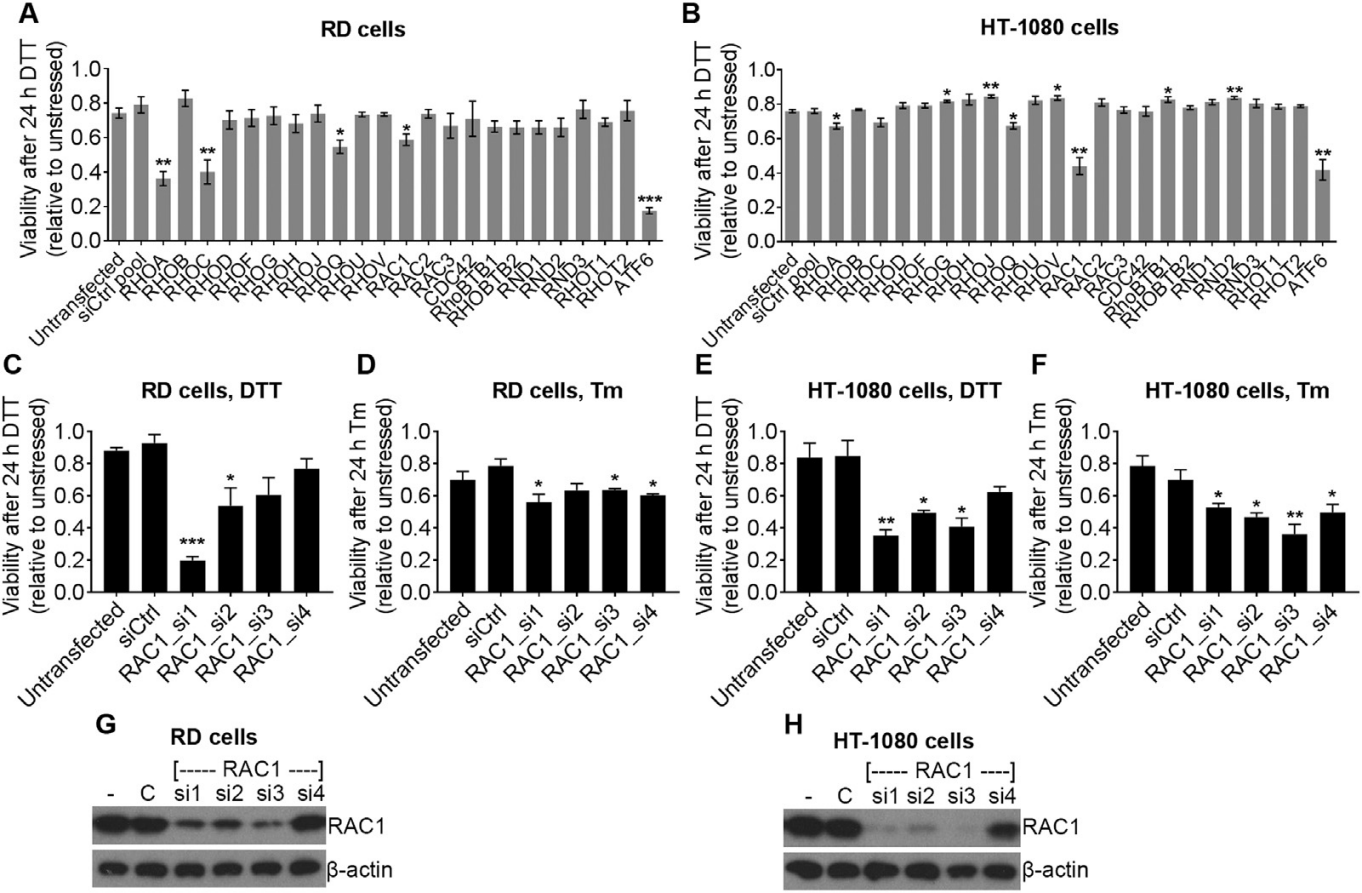
Oncogenic RAC1 protects HT-1080 human fibrosarcoma cells from ER-stress. **A** and **B**, Relative viability (WST-1 assay) of RD cells (**A**) and HT-1080 cells (**B**) after knockdown using pools of four siRNA against the indicated targets followed by treatment with 2 mM DTT for 24 h. Data show relative viability of DTT-treated compared to untreated cells for each siRNA. **C**–**F**, Relative viability (WST-1 assay) of RD cells (**C** and **D**) and HT-1080 cells (**E** and **F**) after knockdown using the indicated individual siRNA followed by treatment with 2 mM DTT (**C** and **E**) or 20 μg/ml Tm (**D** and **F**) for 24 h. Data show relative viability of ER-stressed (DTT or Tm) compared to unstressed cells for each siRNA. **G** and **H**, Representative western blots showing expression of RAC1 and β-actin loading control in RD cells (**G**) or HT-1080 cells (**H**) after knockdown using the indicated siRNA oligomers (- = untransfected, C = siCtrl). Western blots are representative of three independent experiments. Bar charts show means ± S.E.M. of data from three independent experiments. * p < .05, ** p < .01, *** p < .001, unpaired *t*-test comparing to siCtrl, n = 3.

Cells were transfected at the time of seeding using Lipofectamine RNAiMAX (ThermoFisher Scientific) at a final ratio of 0.5 μl RNAiMAX:600 μl medium. siRNA was used at a concentration of 2 nM for pools and single oligomers, and 2 nM per oligomer for combinations of two oligomers. Downstream experiments were performed 48 h after transfection. siRNA was obtained from Qiagen (siCtrl) or Dharmacon (all other siRNA). Product numbers for pools of four oligos (Used for Fig. 1A) are as follows: *RHOA*, M-003860-03; *RHOB*, M-008395-04; *RHOC*, M-008555-01; *RHOD*, M-008940-00; *RHOF*, M-008316-00; *RHOG*, M-008995-00; *RHOH*, M-008804-00; *RHOJ*, M-010367-01; *RHOQ*, M-009943-01; *RHOU*, M-009882-00; *RHOV*, M-006374-02; *RAC1*, M-003560-06; *RAC2*, M-007741-01; *RAC3*, M-008836-02; *Cdc42*, M-005057-01; *RHOBTB1*, M-009389-00; *RHOBTB2*, M-009252-00; *RND1*, M-008929-01; *RND2*, M-009727-01; *RND3*, M-007794-02; *RHOT1*, M-010365-01; *RHOT2*, M-008340-01; *ATF6*, M-009917-01; siCtrl pool, D-001206-14. Product numbers for individual oligomers are as follows: *RAC1*_si1, D-003560-07; *RAC1*_si2, D-003560-08; *RAC1*_si3, D-003560-09; *RAC1*_si4, D-003560-30; *PERK*_si1, D-004883-02; *PERK*_si2, D-004883-05; *ATF4*_si1, D-005125-01; *ATF4*_si2, D-005125-07; *ATF4*_si3, D-005125-08; *ATF4*_si4, D-005125-09; *NRAS*_si1, D-003919-01; *NRAS*_si2, D-003919-02; *NRAS*_si3, D-003919-03; *NRAS*_si4, D-003919-04; siCtrl, SI03650318 (Qiagen).

### 2.3 Inhibitors

The following inhibitors were purchased from Selleck Chemicals, catalogue numbers as follows: GSK2656157 (hearefter referred to as PERKi), S7033; pictilisib (GDC-0941), S1065; AZD8055, S1555; apitolisib (GDC-0980), S2696; selumetinib (AZD6244), S1008; GDC-0879, S1104; RO5126766, S7170; SCH772984, S7101. AKT inhibitor VIII (AKTi VIII) was purchased from Merck Millipore, catalogue number 124018. Concentrations were selected from a dilution series performed to ensure activity. Concentrations used for all experiments are shown in Table 1. Cells were treated with inhibitor 1 h prior to induction of ER-stress.

**Table 1:** Inhibitors, major targets and concentrations used in this study.

Inhibitor	Major target(s)	Concentration	Reference
GSK2656157	PERK	50 and 500 nM	[42]
Pictilisib (GDC-0941)	PI3K	500 nM	[43]
AKT inhibitor VIII	AKT PH domain	5 μM	[44]
AZD8055	MTOR	500 nM	[45]
Apitolisib (GDC-0980)	PI3K and MTOR	500 nM	[46]
Selumetinib (AZD6244)	MEK1	50 nM	[47]
GDC-0879	BRAF/CRAF	50 nM	[48]
RO5126766	BRAF/CRAF/MEK	50 nM	[49]
SCH772984	ERK1/2	50 nM	[50]

### 2.4 Western blotting

For western blotting, cells were lysed in buffer containing 1% Triton X-100, 1% sodium dodecyl sulfate, 150 mM NaCl, 5 mM EDTA, 10 mM Tris, pH 7.6, Complete protease inhibitor (Roche) and PhosStop phosphatase inhibitor (Roche). Protein concentration was measured by bicinchronic assay (ThermoFisher Scientific). 5× Reducing sample buffer (300 mM Tris pH 6.8; 50% v/v glycerol; 2.5 mg/ml bromophenol blue; 250 mg/ml dithiothreitol) was added and lysates were diluted to equal concentrations. Proteins were separated by SDS-PAGE and transferred onto PVDF membrane. Membranes were blocked in Tris-buffered saline containing 5% bovine serum albumin for at least 30 min, then incubated with antibody overnight at 4 °C. Antibodies used were as follows: Cell Signaling Technology: PARP1 (#9532), caspase-3 (#9665), PERK (#3192), phosphorylated EIF2S1 (Serine 51) (#9721), EIF2S1 (#5324), ATF4 (#11815), phosphorylated AKT (Serine 473) (#9271), AKT (#4691), phosphorylated MEK1/2 (Serine 217/221) (#9154), MEK1/2 (#8727), phosphorylated ERK1/2 (Threonine 202/Tyrosine 204) (#4376) and ERK1/2 (#9102). Millipore: NRAS (OP25-100UG) and RAC1 (05-389). ATF6 (ab122897) from Abcam and β-actin (A5441) from Sigma-Aldrich.

### 2.5 Antibody arrays

PathScan Stress and Apoptosis Signalling Antibody Array Kits were purchased from Cell Signaling Technology, product #12923. Cells were lysed and processed for arrays according to the manufacturer's protocol. The protein concentration of the lysate was 0.8 mg/ml and incubations were carried out overnight at 4 °C. Arrays were imaged using an Odyssey Fc imaging system and quantified using Image Studio software (LI-COR).

### 2.6 RNA extraction and quantitive PCR (qPCR)

RNA was extracted and purified from cells using an RNeasy Plus Mini kit (Qiagen) according to the manufacturer's protocol. The resulting RNA was used to produce cDNA using qScript cDNA Supermix (Quantabio). The resulting cDNA was used as a template for TaqMan real-time PCR assays to determine transcript expression. Reaction volume was 20 μl. Glyceraldehyde-3-phosphate dehydrogenase (*GAPDH*) was used as a housekeeping control for relative quantification. The following TaqMan assays were used: *GAPDH*, Hs02758991_g1; *XBP1u*, Hs02856596_m1; *XBP1s*, Hs03929085_g1; *DDIT3*, Hs00358796_g1; *ATF4*, Hs00909569_g1; *PPP1R15A*, Hs00169585_m1; Relative quantity of transcript was quantified by normalising first to *GAPDH*, and then to the measurement from untreated cells using Applied Biosystems 7500 software.

### 2.7 WST-1 cell viability assays and measurement of sensitivity to stress

Cells were seeded in 96-well plates and treated/transfected as indicated in triplicate wells for each independent experiment. Cells were subjected to ER-stress for 24 h (or left unstressed), then washed in medium, and fresh phenol-red-free medium containing WST-1 reagent (Abcam) was added. Cells were subsequently maintained for 1 h prior to measuring absorbance at 440 nM. The negative control background reading was subtracted from all measurements. To measure sensitivity to stress, ‘Viability after 24 h DTT (relative to unstressed)’ for each sample was used i.e. for each siRNA-treatment or compound treatment, unstressed and stressed cell viability was measured by WST-1 assay, then the ratio of the stressed to unstressed measurement for each sample was calculated and is shown in Fig. 1, Fig. 3, Fig. 4, Fig. 5, Fig. 6.

## 3 Results

### 3.1 RAC1 knockdown sensitises RD and HT-1080 cells to ER-stress

Cancer cells are able to resist stress caused by oncogenic transformation and unfavourable micro-environmental conditions. These conditions can induce ER-stress which activates the UPR and can lead to apoptosis if not resolved [2]. Rho GTPases have been linked to evasion of apoptosis [11] and there is evidence that they may be involved in survival under ER-stress in *C. elegans* [19]. In addition, several Rho GTPases bear oncogenic mutations in cancer [11]. We hypothesised that human Rho GTPases may be involved in cell survival under ER-stress and oncogenic mutation of Rho GTPases may protect cells from ER-stress. We devised a strategy to test this using an siRNA screening approach in two different human soft-tissue sarcoma cell lines: RD cells which have wild-type Rho GTPases and HT-1080 cells which contain an oncogenic N92I RAC1 mutation [23]. Both these cell lines also contain a Q61 NRAS mutation. Because the N92I RAC1 mutation is activating, we would expect it to have a similar effect to the P29S mutation in melanoma. Cells were transfected with pools of siRNA targeting all 20 Rho GTPases plus the mitochondrial Rho GTPases RHOT1 and RHOT2. ATF6 is an important pro-survival component of the UPR [8], so ATF6 siRNA was used as a positive control for increased sensitivity to ER-stress. Non-targeting control siRNA (siCtrl pool) was used as a negative control. To induce ER-stress, cells were treated with 2 mM dithiothreitol (DTT) which interferes with disulphide formation within the ER, leading to ER-stress and UPR activation. It should be noted that several siRNA pools affected cell viability in unstressed conditions (Supplementary Fig. S1A and S1B). Therefore, we calculated relative viability compared to unstressed cells for each siRNA to assess sensitivity to stress. In both cell lines, the positive control ATF6 siRNA sensitised cells to ER-stress, seen as lower relative viability after DTT treatment (Fig. 1A and B). In RD cells (wild type GTPases), siRNA pools targeting RHOA, RHOC RHOQ and RAC1 significantly sensitised cells to DTT treatment, with RHOA and RHOC having the strongest effect (Fig. 1A). In HT-1080 cells (N92I RAC1), while pools of siRNA against RHOA and RHOQ had a small but significant effect on sensitivity to ER-stress, siRNA against RAC1 had the strongest effect and was comparable to the ATF6 positive control (Fig. 1B). These results suggest that RHOA, RHOC, RHOQ and RAC1 may be involved in cell survival under ER-stress in wild-type cells, while oncogenic RAC1 mutation may overcome this in HT-1080 cells where RAC1 is the predominant Rho GTPase involved in ER-stress resistance. The observation that oncogenic RAC1 promotes resistance to ER-stress could be important for cancer treatment because, targeting oncogenic RAC1 signalling may specifically target cancer cells over wild-type cells. For this reason, we chose to focus our research on the role of RAC1.

In order to validate the results from the screen, single siRNA oligomers were used and cells were treated with two different ER-stress inducers: 2 mM DTT (as for the screen) or 20 μg/ml tunicamycin (Tm), which induces ER-stress by inhibiting N-linked protein glycosylation leading to a build-up of incorrectly processed protein within the ER. Single oligomers affected cell viability in unstressed cells (Supplementary Fig. S1C and S1D), so viability relative to unstressed cells for each single oligomer was used to assess sensitivity to stress. In RD cells, RAC1_si1 and RAC1_si2 significantly sensitised cells to DTT treatment (Fig. 1C), and RAC1_si1, RAC1_si3 and RAC1_si4 slightly (but significantly) sensitised cells to Tm treatment (Fig. 1D). Results in RD cells did not directly correlate with RAC1 expression as RAC1_si1, RAC1_si2 and RAC1_si3 all knocked down the protein level to a similar level but RAC1_si4 had a weaker effect (Fig. 1G). In HT-1080 cells, three out of four oligomers significantly increased sensitivity to DTT (Fig. 1E), and all oligomers significantly increased sensitivity to Tm (Fig. 1F). The three oligomers that consistently induced sensitivity to ER-stress (RAC1_si1, RAC1_si2 and RAC1_si3) also corresponded to the strongest knockdown in protein expression observed by western blot (Fig. 1H). These observations agree with the siRNA screen and suggest that oncogenic N92I RAC1 in HT-1080 cells promotes resistance to ER-stress. To investigate the mechanism of this, we focussed attention on HT-1080 cells and used two single siRNA oligomers for RAC1 knockdown (RAC1_si1 and RAC1_si2) in subsequent experiments.

**Fig. 2:**
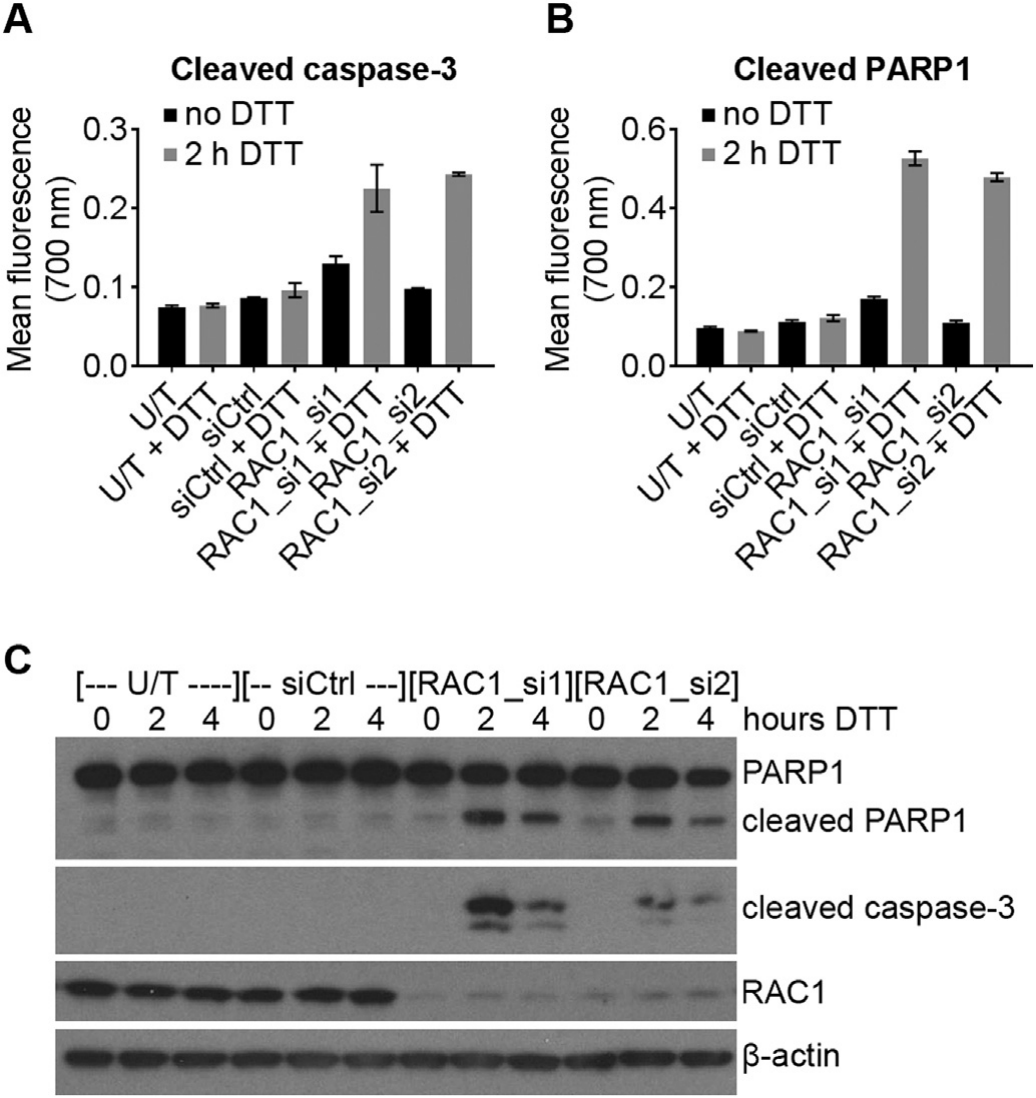
RAC1 knockdown in HT1080 human fibrosarcoma cells causes increased caspase activity under ER-stress. **A** and **B**, Mean ± standard deviation fluorescence readings from an ELISA-based antibody array measuring cleaved caspase-3 (**A**) and cleaved PARP1 (**B**) in lysates from HT-1080 cells transfected with the indicated siRNA oligomers, followed by treatment with 2 mM DTT for 2 h (grey bars) or no treatment (black bars). **C**, Representative western blots showing expression of PARP/cleaved PARP, cleaved caspase-3, RAC1 and β-actin loading control in lysates from HT-1080 cells transfected with the indicated siRNA, followed by treatment with 2 mM DTT for 0, 2 or 4 h. Western blots are representative of three independent experiments. U/T = untransfected.

The WST-1 assay uses reduction of the WST-1 reagent as a measure of cell viability, but does not directly measure cell death. Our next step was to confirm that RAC1 knockdown leads to increased apoptosis under ER-stress by measuring cleavage of Poly [ADP Ribose] Polymerase 1 (PARP1) and caspase-3, which can be used as a readout of apoptosis [24]. We first used a sandwich ELISA-based antibody array to probe cell stress and apoptosis signalling pathways. Arrays detected increased cleavage of PARP1 and caspase-3 in RAC1-depleted cells under ER-stress compared to data from untransfected and siCtrl-transfected cells (Fig. 2A and B), indicating that more apoptosis occurred in RAC1-depleted cells under ER-stress. To confirm these results, cleavage of PARP1 and caspase-3 were measured in cell lysates. Western blots confirmed that cleavage of PARP1 and caspase-3 occurred more strongly under ER-stress in RAC1-depleted cells than in siCtrl or untransfected cells (Fig. 2C). These data confirm that RAC1 knockdown in HT-1080 cells increases sensitivity to ER-stress by increasing apoptosis.

### 3.2 RAC1 knockdown alters the UPR in HT-1080 cells

**Fig. 3:**
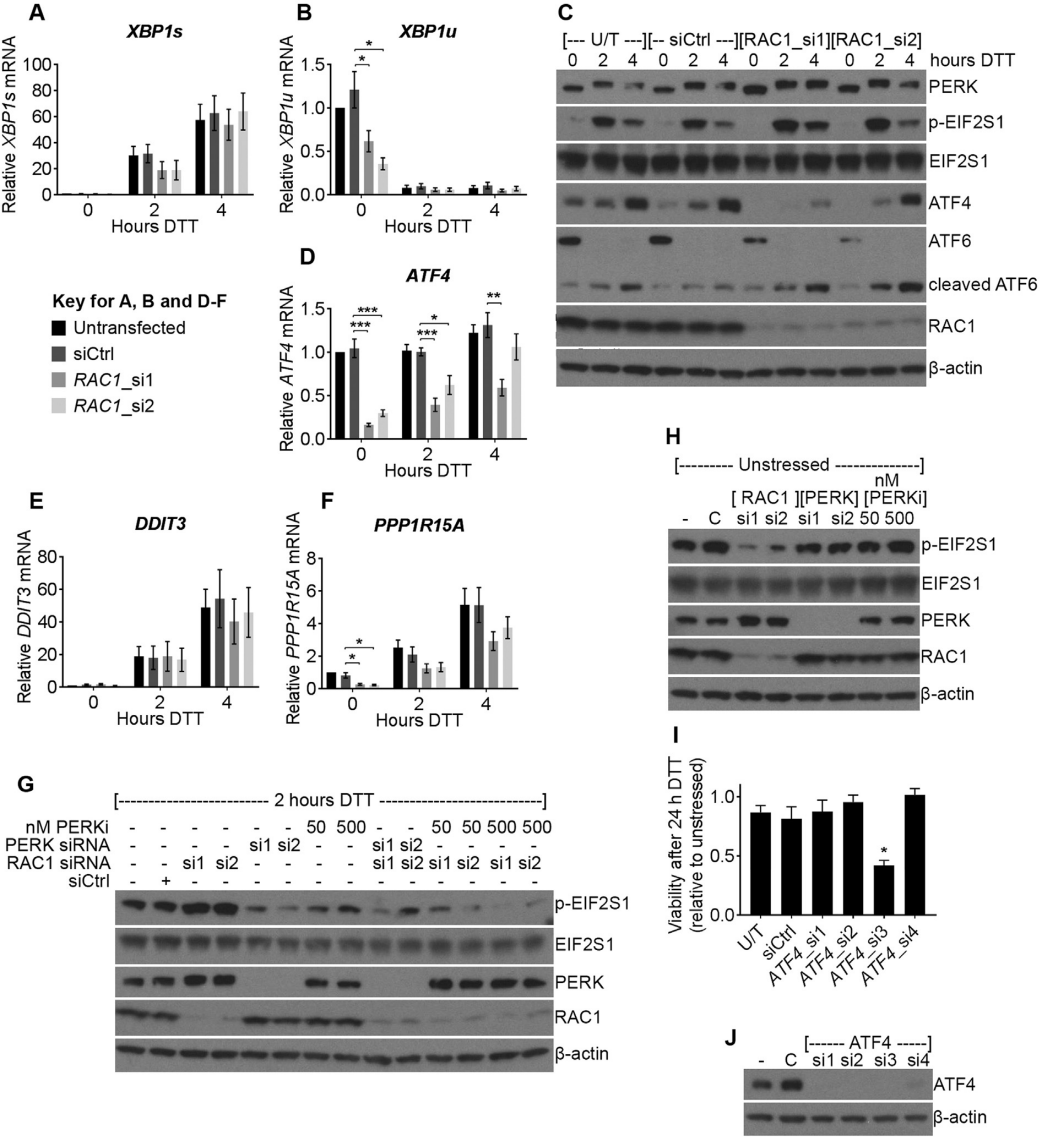
RAC1 knockdown alters the UPR in HT-1080 human fibrosarcoma cells. **A**, **B** and **D**–**F**, mRNA expression of the indicated transcripts (TaqMan qPCR) in HT-1080 cells transfected with the indicated siRNA and treated with 2 mM DTT for the indicated times. **C**, western blots showing expression or phosphorylation of the indicated proteins in lysates from HT-1080 cells treated as in A (U/T = untransfected). **G** and **H**, western blots showing phosphorylation or expression of the indicated proteins in lysates from HT-1080 cells transfected with the indicated siRNA or treated with the indicated concentration (nM) of GSK2656157 (PERKi) for 1 h, then either stressed with 2 mM DTT for 2 h (**G**) or kept unstressed (**H**). **I**, Relative viability (WST-1 assay) of HT-1080 cells transfected with the indicated siRNA targeting ATF4 and treated with 2 mM DTT for 24 h. Data show relative viability of DTT-treated compared to untreated cells for each siRNA. **J**, Western blots showing expression of ATF4 and β-actin loading control in lysates from HT-1080 cells transfected with the indicated siRNA (- = untransfected, C = siCtrl). Western blots are representative of at least three independent experiments. Bar charts show means ± S.E.M. of data from three independent experiments. * p < .05, ** p < .01, *** p < .001, unpaired *t*-test n = 3.

Because cell fate under ER-stress is determined by the UPR [2], we next sought to determine whether RAC1 knockdown in HT-1080 cells influenced activation of the UPR. The UPR consists of IRE1/XBP1, ATF6 and PERK/EIF2S1/ATF4 arms [9]. To measure activation of the IRE1 arm of the UPR we used TaqMan quantitative PCR (qPCR) to measure *XBP1s* and *XBP1u* transcripts as previously described [3]. As expected, in untransfected and siCtrl cells, splicing of *XBP1* mRNA was induced by DTT treatment, observed as an increase in *XBP1s* (Fig. 3A) and decrease in *XBP1u* (Fig. 3B). Knockdown of RAC1 reduced the expression of *XBP1u* (Fig. 3B), leading to a slight reduction in *XBP1s* at 2 h post-DTT treatment, but this was not statistically significant (Fig. 3A). This suggests that oncogenic RAC1 may increase expression of *XBP1u* in unstressed HT-1080 cells.

ATF6 activation was measured by observing the appearance of the cleaved form of ATF6 by western blotting. ATF6 was cleaved after DTT treatment in all samples, but the expression of cleaved ATF6 after DTT treatment was higher in RAC1-depleted cells compared to controls (Fig. 3C).

To measure activation of the PERK UPR arm, we used western blotting to observe electrophoretic mobility shift of activated PERK, phosphorylation of EIF2S1 at Serine 51 (a PERK substrate) and expression of ATF4 protein (downstream of EIF2S1 phosphorylation). PERK was activated after DTT treatment (observed as an electrophoretic mobility shift to a slower band on the western blot) and this was not affected by RAC1 depletion compared to control cells (Fig. 3C), although RAC1 knockdown induced an increase in total PERK protein expression observed (Fig. 3C). Phosphorylation of EIF2S1 and expression of ATF4 protein both increased after DTT treatment in untransfected and siCtrl-transfected cells, and RAC1 knockdown altered this response as follows: in unstressed conditions (0 h DTT), RAC1 knockdown led to a reduction in phosphorylated EIF2S1 (p-EIF2S1) and a corresponding reduction in ATF4 compared to untransfected and siCtrl cells (Fig. 3C). However, after DTT treatment p-EIF2S1 was higher in RAC1-depleted cells compared to untransfected and siCtrl-transfected cells, especially at the 2 h time-point, whereas ATF4 expression remained impaired (Fig. 3C).

The observation of higher p-EIF2S1, but lower ATF4 expression in RAC1-depleted cells after 2 h DTT treatment is seemingly counterintuitive. We would expect ATF4 expression to increase with EIF2S1 phosphorylation [9]. A possible explanation for this discrepancy would be if the level of *ATF4* mRNA was reduced, so we measured *ATF4* mRNA expression by qPCR in samples from RAC1-depleted and control cells, with or without DTT treatment. In untransfected or siCtrl-transfected cells, DTT treatment did not affect the expression of *ATF4* mRNA (Fig. 3D). In RAC1-depleted cells, there was a significant reduction in ATF4 mRNA in unstressed conditions (0 h DTT), and the ATF4 mRNA level became responsive to ER-stress—increasing with DTT treatment (Fig. 3D). These data explain the observation that ATF4 protein expression is lower in RAC1-depleted cells despite the presence of increased p-EIF2S1 after DTT treatment. They also suggest that N92I RAC1 may drive overexpression of *ATF4* mRNA in basal conditions, because *ATF4* transcript only responded to ER-stress when RAC1 was depleted.

In the UPR, ATF4 activates transcription of *DDIT3*, a key pro-apoptotic transcription factor that executes apoptosis caused by an unresolved UPR [9]. We therefore tested whether RAC1 knockdown leads to an inhibition of *DDIT3* induction. qPCR measurements showed that DTT treatment led to an increase in *DDIT3* mRNA expression which was not significantly affected by the knockdown of RAC1 (Fig. 3E), suggesting that *DDIT3* is not part of the mechanism for the increased sensitivity of RAC1-depleted cells to ER-stress.

We next investigated the mechanism for the observed modulation of p-EIF2S1 caused by RAC1 knockdown. There is a known negative feedback loop controlled by ATF4, which promotes the expression of protein phosphatase 1 regulatory subunit 15A (PPP1R15A, also known as GADD34) [25]. PPP1R15A activates serine/threonine phosphatase PP1 to dephosphorylate EIF2S1 [26]. We hypothesised that the reduced *ATF4* mRNA level observed in RAC1-depleted cells may down-regulate this negative feedback loop leading to EIF2S1 hyper-phosphorylation upon ER-stress. To test this hypothesis, we first measured the expression of *PPP1R15A* in RAC1-depleted HT-1080 cells by qPCR. Compared to siCtrl, RAC1 knockdown led to a significant reduction of *PPP1R15A* in unstressed cells which recovered after DTT treatment (Fig. 3F) correlating with the data for *ATF4* mRNA (Fig. 3D). This shows that the loss of RAC1 in HT-1080 cells depletes a controlling component of the negative-feedback loop from ATF4 to EIF2S1 in resting conditions. *PPP1R15A* expression was induced after ER-stress in all samples (Fig. 3F), which would explain the peak of EIF2S1 at 2 h DTT followed by dephosphorylation at 4 h as the negative feedback loop returned (Fig. 3C). We hypothesised that RAC1-depleted cells may therefore be primed for hyper-phosphorylation by PERK upon activation of the UPR. If this is correct, inhibition of PERK should prevent the hyper-phosphorylation of EIF2S1. To test this, we used combined knockdown of RAC1 and PERK, or RAC1 knockdown combined with the PERK inhibitor GSK2656157 (hereafter termed PERKi). Knockdown of RAC1 led to hyper-phosphorylation of EIF2S1 compared to control cells as before (Fig. 3G), and knockdown or inhibition of PERK reduced EIF2S1 phosphorylation and inhibited the hyper-phosphorylation induced by RAC1-depletion (Fig. 3G). These data agree with the hypothesis that RAC1 knockdown impairs negative feedback on EIF2S1 phosphorylation leading to hyper-phosphorylation in early stages of ER-stress.

It might be expected that basal EIF2S1 phosphorylation would be higher upon loss of the ATF4/PP1 feedback loop. However, basal EIF2S1 phosphorylation was lower in RAC1-depleted cells (Fig. 3C). To test whether the loss of PERK activity may be responsible for basal EIF2S1 phosphorylation, we used siRNA and PERK inhibition. As seen in Fig. 3C, RAC1 knockdown caused a reduction of p-EIF2S1 in these unstressed conditions (Fig. 3H). However, EIF2S1 phosphorylation was not affected by PERK knockdown or small molecule inhibition suggesting that PERK is not responsible for basal EIF2S1 phosphorylation in HT-1080 cells.

Taken together, these data show that RAC1 depletion in HT-1080 cells alters UPR function: Under basal conditions, knockdown of RAC1 leads to a reduction in *XBP1u* and *ATF4* mRNA, together with reduced ATF4 protein and decreased phosphorylation of EIF2S1. After DTT treatment, compared to control cells, RAC1-depleted HT-1080 cells have lower *ATF4* mRNA and protein, and a higher level of cleaved ATF6, PERK protein and p-EIF2S1. We next investigated whether these modulations of the UPR may cause increased sensitivity to ER-stress. There is data already available for the roles of XBP1, PERK and ATF6 in ER-stress in HT-1080 cells. We have previously shown that inhibition of *XBP1* splicing does not significantly affect HT-1080 cell survival under DTT treatment [3]. In the present work we show that ATF6 knockdown induces sensitivity to DTT treatment, (Fig. 1B) so we would not expect increased ATF6 cleavage in RAC1-depleted cells to cause sensitivity to ER-stress. In the case of PERK, inhibition of this kinase (and therefore inhibition of ER-stress-induced EIF2S1 phosphorylation) has been shown to sensitise HT-1080 cells to ER-stress [27]. Hence, we would not expect EIF2S1 hyper-phosphorylation to increase sensitivity to ER-stress. Therefore, we sought to determine whether reduced ATF4 signalling may cause the enhanced sensitivity to ER-stress in RAC1-depleted cells. To do this, we tested whether knockdown of ATF4 would mimic the effect of RAC1 knockdown on cell viability under ER-stress. Of the four siRNA oligomers targeting ATF4 tested, only one caused a significant decrease in cell viability after DTT treatment (Fig. 3I), whereas all oligomers induced protein knockdown (Fig. 3J). This suggests an off-target effect of the oligomer ATF4_si3. Taken together with the previous evidence using inhibitors of IRE1 and PERK [3, 27], the present data from ATF6 knockdown (Fig. 1B) and ATF4 silencing (Fig. 3I) suggest that despite the presence of an altered UPR in RAC1-depleted HT-1080 cells, this is probably not the mechanism for their increased sensitivity to ER-stress.

Because RAC1 is a regulator of the oncogenic and cell-protective kinase signalling pathways PI3K/AKT/MTOR and RAF/MEK/ERK, we next sought to determine how signalling through these pathways may play a role in ER-stress resistance.

### 3.3 Inhibition of MEK/ERK signalling causes sensitivity to ER-stress in HT-1080 and RD cells

**Fig. 4:**
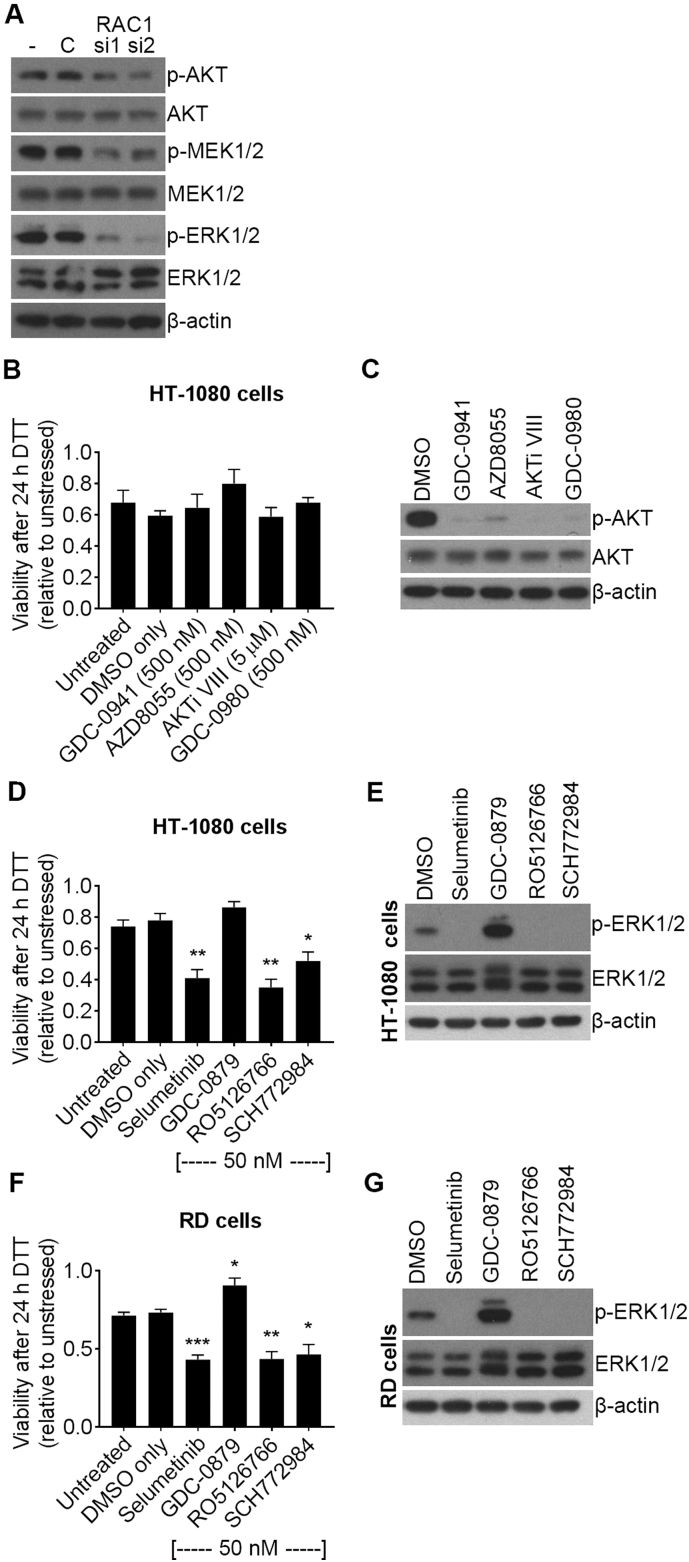
Inhibition of MEK or ERK, but not PI3K, AKT or MTOR, causes sensitivity to ER-stress in HT-1080 human fibrosarcoma cells. **A**, Western blots showing expression or phosphorylation of the indicated proteins in lysates from HT-1080 cells transfected with the indicated siRNA (- = untransfected, C = siCtrl). **B**, **D** and **F**, Relative viability measurements (WST-1 assay) of HT-1080 cells (**B** and **D**) or RD cells (**F**) treated with the indicated inhibitors for 1 h, then 2 mM DTT for 24 h. Data show relative viability of DTT-treated compared to untreated cells for each inhibitor. **C**, **E** and **G**, Western blots showing expression and phosphorylation of the indicated proteins in lysates from HT-1080 cells (**C** and **E**) or RD cells (**G**) treated with the indicated inhibitors, or DMSO vehicle control, for 1 h. Western blots are representative of at least three experiments. Bar charts show means and S.E.M. of data from three independent experiments. * p < .05, ** p < .01, *** p < .001, unpaired *t*-test comparing to DMSO only, n = 3.

RAC1 has previously been linked to PI3K/AKT/MTOR and RAF/MEK/ERK signalling [12, 14, 15] and both these pathways can be important mechanisms of cancer cell evasion of apoptosis [16]. We hypothesised that N92I RAC1 may drive anti-apoptotic signalling through PI3K/AKT/MTOR and RAF/MEK/ERK in HT-1080 cells, leading to resistance to ER-stress. To test this, we first determined whether RAC1 knockdown leads to a reduction in the activity, and hence phosphorylation of AKT, MEK1/2 or ERK1/2. Knockdown of RAC1 in HT-1080 cells led to reduced phosphorylation of AKT^SER473^ (p-AKT), MEK1/2^SER217/221^ (p-MEK1/2) and ERK1/2^THR202/TYR204^ (p-ERK1/2) compared to control cells (untransfected and siCtrl) (Fig. 4A). This suggests that oncogenic RAC1 contributes to constitutive activation of AKT and MEK/ERK in HT-1080 cells. To test whether reduced AKT or MEK/ERK signalling may drive resistance to ER-stress, we used a panel of clinically relevant small molecule inhibitors (Table 1). HT-1080 cells were treated with each inhibitor for 1 h prior to treatment with DTT for 24 h. Inhibitor remained present throughout the experiment and cell viability was measured by WST-1 assay as before. PI3K/AKT/MTOR pathway inhibitors had no significant effect on sensitivity to ER-stress (Fig. 4B), despite strong inhibition of AKT phosphorylation (Fig. 4C) and significant effects on cell viability in unstressed conditions (Supplementary Fig. S2A). In the case of RAF/MEK/ERK inhibitors, selumetinib, RO5126766 and SCH772984, all reduced relative cell viability after DTT treatment compared to DMSO vehicle control (Fig. 4D). GDC-0879 slightly increased relative viability after ER-stress but this was not significant (Fig. 4D). Interestingly, these results correlated with inhibition of ERK phosphorylation: selumetinib, RO5126766 and SCH772984 all potently inhibited phosphorylation of ERK1/2, but GDC-0879 caused paradoxical activation of ERK1/2 (Fig. 4E). The phenomenon of paradoxical ERK1/2 activation caused by GDC-0879 activating wild type RAF has been previously described in other cell lines [22]. RAF/MEK/ERK inhibition had no effect on HT-1080 cell viability in unstressed conditions (Supplementary Fig. S2B). These results indicate that MEK/ERK signalling may protect cells from ER-stress. To confirm that this effect is not unique to HT-1080 cells, we tested the effect of RAF/MEK/ERK inhibition on cell viability under ER-stress in RD cells. RD cells express oncogenic Q61H NRAS which would be expected to drive RAF/MEK/ERK activity. We treated RD cells in the same way as HT-1080 cells for Fig. 4D and E and measured viability after DTT treatment, and ERK1/2 phosphorylation as before. Data from RD cells matched those from HT-1080 cells: Western blots showed the same expected paradoxical ERK1/2 phosphorylation after GDC-0879 treatment while the other inhibitors all strongly inhibited ERK1/2 (Fig. 4G). Correlating with this, selumetinib, RO5126766 and SCH772984 all reduced relative cell viability after DTT treatment compared to DMSO vehicle control, and GDC-0879 slightly increased relative viability after ER-stress (Fig. 4F). RAF/MEK/ERK inhibition had a weak effect on RD cell viability in unstressed conditions (Supplementary Fig. S2C). These data indicate that constitutive ERK activation in cancer cells drives resistance to ER-stress.

### 3.4 Paradoxical ERK activation by RAF inhibition in NRAS-mutant cells partially rescues the effect of RAC1 knockdown on ER-stress

**Fig. 5:**
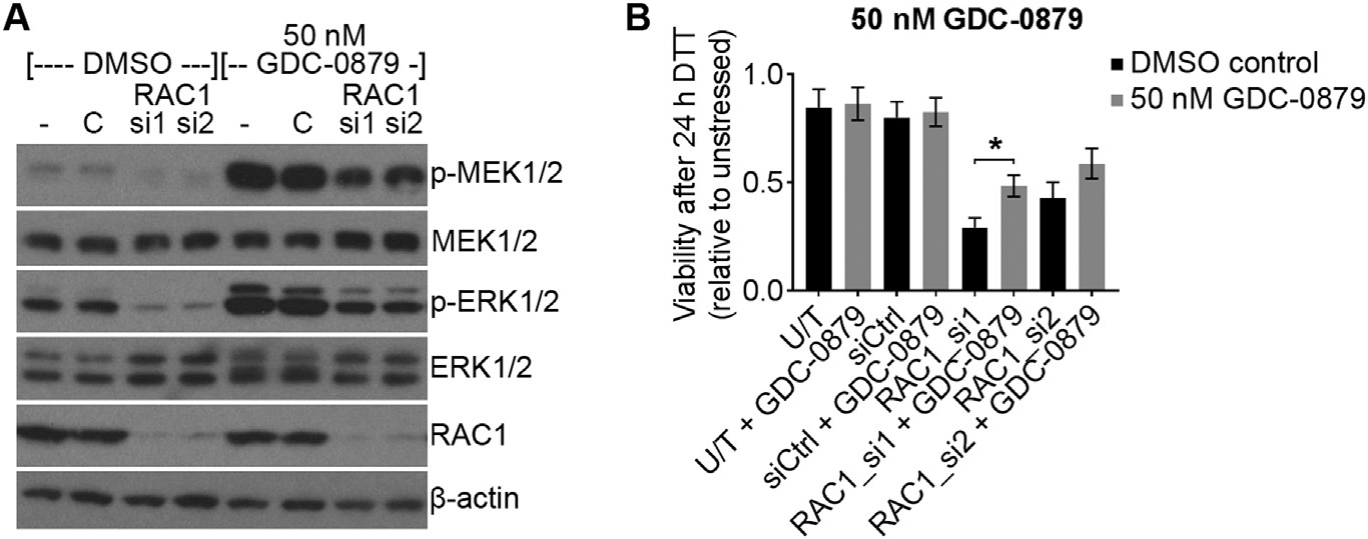
ERK activation induced by the BRAF/CRAF inhibitor GDC-0879 partially rescues the effect of RAC1 knockdown on ER-stress sensitivity. **A**, Western blots showing phosphorylation and expression of the indicated proteins in lysates from HT-1080 cells transfected with the indicated siRNA for 48 h followed by 50 nM GDC-0879 or DMSO vehicle control for 1 h (- = untransfected, C = siCtrl). **B**, Relative viability measurements (Wst-1 assay) of HT-1080 cells transfected with the indicated siRNA for 48 h, then treated with 50 nM GDC-0879 (grey bars) or DMSO vehicle control (black bars) for 1 h, then 2 mM DTT for 24 h. Data show relative viability of DTT-treated compared to untreated cells for each inhibitor or siRNA treatment (U/T = untransfected). Western blots are representative of three experiments. Bar charts show means ± S.E.M. of data from three independent experiments. * p < .05, unpaired *t*-test n = 3.

Our results suggest that N92I RAC1 drives resistance to ER-stress by increasing MEK/ERK activity. We therefore hypothesised that the paradoxical ERK activation caused by GDC-0879 may overcome the effect of RAC1 knockdown on sensitivity to ER-stress. To test this, HT-1080 cells were transfected with control or RAC1 siRNA, then treated with GDC-0879 or DMSO vehicle control for 1 h. Western blotting showed that knockdown of RAC1 led to a reduction in p-MEK1/2 and p-ERK1/2, and 50 nM GDC-0879 caused hyper-phosphorylation of MEK1/2 and ERK1/2 in both control and RAC1-depleted cells (Fig. 5A). Measurements of cell viability (WST-1 assay) after 24 h DTT treatment showed that 50 nM GDC-0879 partially rescued the effect of RAC1 knockdown on sensitivity to ER-stress (Fig. 5B). These results confirm the hypothesis that the loss of MEK/ERK activity is at least partly responsible for the effect of RAC1 knockdown on sensitivity to ER-stress in HT-1080 cells.

### 3.5 Knockdown of NRAS induces sensitivity to ER-stress in NRAS mutant cell lines

**Fig. 6:**
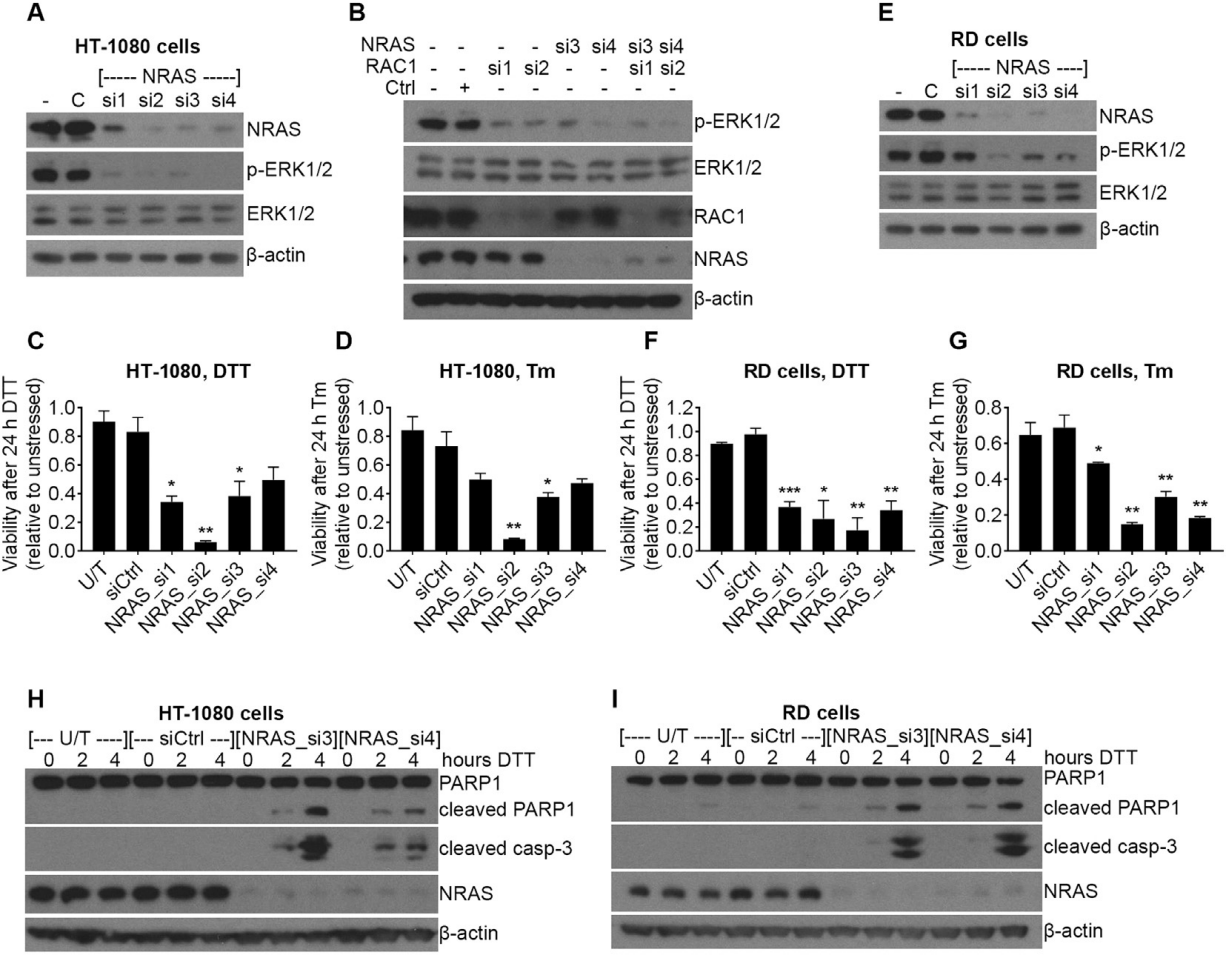
Knockdown of NRAS causes sensitivity to ER-stress in HT-1080 and RD human cancer cell lines. **A** and **E**, western blots showing expression and phosphorylation of the indicated proteins in lysates from HT-1080 cells (**A**) or RD cells (**E**) transfected with the indicated siRNA. **B**, Western blots showing phosphorylation or expression of the indicated proteins in lysates from HT-1080 cells transfected with the indicated siRNA. **C**, **D**, **F** and **G**, Relative viability (WST-1 assay) of HT-1080 cells (**C** and **D**) or RD cells (**F** and **G**) after transfection with the indicated siRNA, followed by treatment with 2 mM DTT (**C** and **F**) or 20 μg/ml Tm (**D** and **G**) for 24 h. Data show mean and S.E.M. of relative viability of stressed (DTT or Tm) compared to unstressed cells for each siRNA. * p < .05, ** p < .01, *** p < .001, unpaired *t*-test comparing to siCtrl, n = 3. **H** and **I**, western blots showing expression of the indicated proteins in lysates from HT-1080 cells (**H**) or RD cells (**I**) transfected with the indicated siRNA, then treated with DTT for the indicated times. U/T = untransfected, western blots are representative of three experiments.

Our data show that RAC1 expression in HT-1080 cells is required for full constitutive activation of the MEK/ERK signalling pathway. A known activator of MEK/ERK signalling in cancer is oncogenic NRAS, which directly activates the kinase RAF upstream of MEK [28]. There is evidence that aberrant signalling and cell proliferation caused by Q61K NRAS in melanocytes is dependent on RAC1 [29]. HT-1080 cells express oncogenic mutants of both RAC1 (N92I) and NRAS (Q61K) [23], so we next sought to determine whether the presence of N92I RAC1 in this cell line would promote the constitutive activation of MEK/ERK regardless of the expression of NRAS. Each of four different siRNA oligomers successfully knocked down NRAS protein and strongly reduced the amount of phosphorylated ERK1/2 (Fig. 6A). We next sought to investigate whether NRAS or RAC1 has a stronger effect on constitutive ERK1/2 activity, or whether the expression of NRAS or RAC1 is dependent on the other. To do this, we co-transfected HT-1080 cells with two different combinations of siRNA targeting both NRAS or RAC1, or the single oligomers alone. Our results showed that RAC1 and NRAS are not dependent on each other for expression, as RAC1 oligomers did not knock down NRAS and vice versa (Fig. 6B). Knockdown of RAC1 or NRAS had a similar effect on the level of p-ERK1/2, as the combined knockdown gave no further reduction compared to single oligomers (Fig. 6B). These data show that both RAC1 (N92I) and NRAS (Q61K) are required for constitutive activation of MEK/ERK signalling in HT-1080 cells. Because we have shown that inhibition of MEK/ERK sensitises HT-1080 cells to ER-stress (Fig. 4), and NRAS knockdown strongly reduced MEK/ERK activity (Fig. 6A), we hypothesised that NRAS knockdown would make HT-1080 cells more sensitive to ER-stress. We tested this by using siRNA to reduce NRAS expression in HT-1080 cells, then treating cells with two different ER-stressors (2 mM DTT or 20 μg/ml Tm) for 24 h. For both stressors, all siRNA oligomers reduced the viability of stressed cells compared to unstressed, and this was significant for three (DTT treatment) or two (Tm treatment) oligomers (Fig. 6C and D). The RD cell line expresses a Q61H mutant NRAS which we would expect to drive ERK1/2 activity. Therefore, we tested whether NRAS knockdown in RD cells would lead to a reduction in ERK1/2 activity combined with increased sensitivity to ER-stress. RD cells were treated in the same way as the HT-1080 cells for Fig. 6A, C and D. Results from RD cells were similar to those from HT-1080 cells. Each siRNA oligomer reduced NRAS protein expression and ERK1/2 phosphorylation (Fig. 6E) and NRAS-depleted cells were also more sensitive to both DTT and Tm treatment, as NRAS-depleted cells had a lower relative viability than control cells (Fig. 6F and G). Interestingly, single siRNA oligomers targeting NRAS had only weak effects on cell viability in the absence of ER-stress suggesting NRAS is not the only driver of HT-1080 or RD cell proliferation (Supplementary Fig. S3A and B). In agreement with cell viability data, knockdown of NRAS caused an increase in apoptosis after DTT treatment of both HT-1080 and RD cells, observed as an increase in cleaved PARP and cleaved caspase-3 (Fig. 6H and I). Together, these results suggest that NRAS drives resistance to ER-stress in Q61 NRAS mutant cell lines. Data from HT-1080 cells show that the presence of N92I RAC1 alone is not sufficient to fully activate MEK/ERK and protect against ER-stress in the absence of Q61K NRAS.

## 4 Discussion

Cancer cells are able to survive under micro-environmental conditions that lead to ER-stress. The mechanisms underlying this are likely to be multifaceted and dependent on the genetic background of the cancer cells. The cell signalling response that controls the balance between cell survival and cell death upon ER-stress is the UPR [9] and previous studies have shown that this plays an important role in cancer cell adaption to stress and cancer development [30]. Many Rho GTPases are strongly linked to cancer through mutation or overexpression [11, 20]. In *C. elegans*, knockout of a Rho GTPase impairs the UPR [19], suggesting that a human Rho GTPase may have a similar role. In the present study, we examined the role of Rho GTPases in cancer cell survival under ER-stress using siRNA screening and in vitro chemical ER-stress inducers. Our siRNA screening results showed that RAC1 was particularly important in cell survival under ER-stress in HT-1080 cells which express an oncogenic RAC1 mutant, compared to RD cells which express wild type RAC1. In RD cells, RHOA and RHOC knockdown had a stronger effect on sensitivity to ER-stress than RAC1 knockdown. Therefore, further study of RHOA and RHOC in the stress response of wild-type cells is warranted. Rho GTPases have been well characterised as modulators of the cytoskeleton, including control of myosin activity [20] and it has been suggested that Myosin IIB controls the clustering of IRE1—a key component of the UPR [21]. It may be the case that RHOA and/or RHOC control the function of Myosin IIB in ER-stress.

Because the UPR is the key response controlling cell fate under ER-stress, we determined the effect of RAC1 depletion on UPR activity in HT-1080 cells. In unstressed conditions, knockdown of RAC1 led to a reduction in *XBP1u* and *ATF4* and *PPP1R15A* mRNA expression. Further work is required to determine the mechanism of this reduction. It is possible that N92I RAC1 drives a regulatory network that controls *XBP1* and *ATF4* transcription. RAC1 expression has previously been linked to transcription factor expression in glioma [31], and while the RNAseq data presented did not link *ATF4* and *XBP1* to RAC1 expression, the association may depend on cell type and genetic background. Our data show that RAC1 knockdown led to PERK-dependent hyper-phosphorylation of EIF2S1 shortly after induction of ER-stress, which is accounted for by impaired expression of the ATF4/PPP1R15A negative feedback loop. It is also possible that the observed increased PERK expression in RAC1-depleted cells contributes to the hyper-phosphorylation. In unstressed conditions, despite the loss of ATF4/PPP1R15, EIF2S1 phosphorylation was lower in RAC1-depleted cells compared to control cells. We show using siRNA and PERK inhibition that PERK is not responsible for basal EIF2S1 phosphorylation in these cells, in agreement with a previous study [27]. These findings imply that a different kinase acts downstream of RAC1 to phosphorylate EIF2S1 in basal conditions. There are three other EIF2S1 kinases in humans: EIF2AK1 (also known as HRI), EIF2AK2 (also known as PKR) and EIF2AK4 (also known as GCN2). A possible candidate for basal phosphorylation in HT-1080 is GCN2, as GCN2 knockout mouse embryonic fibroblasts display reduced EIF2S1 phosphorylation [32]. Although the UPR was altered by RAC1 knockdown, these changes were not associated with the phenotype of increased sensitivity to ER-stress. We showed that the small molecule PERK inhibitor GSK2656157 (PERKi) inhibits EIF2S1 hyper-phosphorylation in RAC1-depleted cells. However, it is known that inhibition of PERK has a sensitising effect rather than a protective effect under ER-stress [27]. In the case of XBP1 splicing, we have previously shown that inhibition of IRE1 does not sensitise HT-1080 cells to DTT treatment [3]. In the present work, we tested the effect of ATF4 knockdown on ER-stress sensitivity and found that there was no consistent significant effect. In addition, DDIT3 expression was not significantly different between RAC1 knockdown and control cells, meaning that the apoptotic signal downstream of the UPR was still active.

**Fig. 7:**
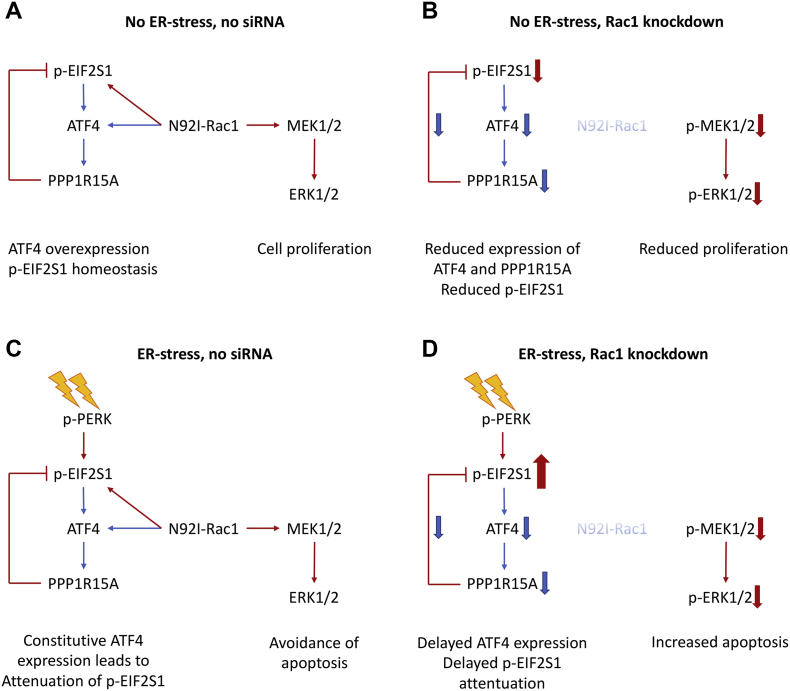
Graphical representation of findings from HT-1080 cells. **A**, In basal conditions, N92I-RAC1 promotes increased p-MEK1/2 and p-ERK1/2 and p-EIF2S1. Overexpression of ATF4 contributes to p-EIF2S1 homeostasis. **B**, Upon RAC1 knockdown, reduced p-MEK1/2 and p-ERK1/2 leads to reduced cell proliferation. There is a loss of basal p-EIF2S1 which is not rescued by the concomitant loss of ATF4 expression. **C**, ER-stress in untransfected cells leads to phosphorylation of EIF2S1 which is quickly attenuated by the N92I-RAC1-driven overexpression of ATF4. N92I-RAC1-dependent p-MEK1/2 and p-ERK1/2 promotes cell survival. **D**, Loss of constitutive ATF4 expression in RAC1-depleted cells leads to hyper-phosphorylation of EIF2S1 by PERK in ER-stressed cells because negative feedback is attenuated. Reduced pMEK1/2 and p-ERK1/2 causes increased sensitivity to stress. Bold arrows represent comparison with corresponding untransfected cells. Red arrows represent phosphorylation, blue arrows represent expression. (For interpretation of the references to colour in this figure legend, the reader is referred to the web version of this article.)

Because the UPR was not responsible for the phenotype of ER-stress-sensitivity of RAC1-depleted cells, we turned our attention to RAC1/cancer-linked kinase signalling pathways PI3K/AKT/MTOR and RAF/MEK/ERK [12, 14, 15]. One function of these signalling pathways is in the avoidance of apoptosis, an important hallmark of cancer [10, 16]. Knockdown of RAC1 led to a reduction in phosphorylation/activity, of AKT, MEK1/2 and ERK1/2 in unstressed conditions. The signalling mechanisms that link RAC1 to AKT, MEK and ERK are not yet fully determined. RAC1 activates p21-activated kinase 1 (PAK1) and it has been proposed that RAC1/PAK1 can promote MEK activity by direct phosphorylation [33] and through a scaffold function [34]. RAC1/PAK1 has also been linked to PI3K and AKT, potentially through reactive oxygen species [35] but a proven mechanism has so far been elusive. The PI3K/AKT/MTOR and RAF/MEK/ERK pathways are important therapeutic targets in cancer and therefore there are numerous clinically important small molecule inhibitors available to examine their function [36]. We used a panel of inhibitors and found that inhibition of MEK/ERK but not PI3K/AKT/MTOR caused sensitivity to ER-stress. Sensitivity to ER-stress correlated with inhibition of ERK1/2 phosphorylation. We observed the expected paradoxical activation of ERK induced by GDC-0879 (in a similar way to that described for other cell lines [22]) and this protected RD cells against ER-stress, and partially rescued the effect of RAC1 knockdown on ER-stress in HT-1080 cells. We conclude that the loss of ERK activity is part of the mechanism for increased sensitivity to ER-stress in RAC1-depleted HT-1080 cells but because this rescue effect was only partial, it is likely that there is also another ER-stress-survival signalling pathway that is perturbed by loss of RAC1. A summary of the effects of N92I-RAC1 knockdown in HT-1080 cells is shown in Fig. 7.

ERK signalling is an important part of the mechanism of oncogenic NRAS, and both HT-1080 cells and RD cells contain an oncogenic Q61 NRAS mutation, so we investigated whether knockdown of NRAS would produce a similar phenotype to knockdown of RAC1 or inhibition of MEK/ERK. Knockdown of NRAS in both HT-1080 and RD cell lines significantly caused sensitivity of cells to ER-stress and a reduction in phosphorylation of MEK1 and ERK1/2. Interestingly, we found that simultaneous knockdown of RAC1 and NRAS in HT-1080 cells did not have a stronger effect on ERK1/2 phosphorylation than either siRNA on their own. This shows that oncogenic RAC1 and NRAS are co-dependent to activate MEK/ERK signalling in HT-1080 cells. In agreement with this, it has previously been reported that signalling and cell growth caused by Q61K NRAS in melanocytes is dependent on RAC1 [29]. The mechanism for this co-dependence remains to be determined. In RD cells, RAC1 knockdown had a weaker effect on sensitivity to ER-stress than NRAS knockdown. This suggests that NRAS may not depend on RAC1 in this cell line (although efficiency of knockdown may have an effect). Instead, RHOA, RHOC, and to a lesser extent RHOQ significantly reduced viability under ER-stress in RD cells so it would be of interest to determine whether one of these affects ERK phosphorylation and cooperates with NRAS. A connection between NRAS and RHOA is not unprecedented, as loss of NRAS in C-cell thyroid adenomas in mice leads to increased RHOA activity [37].

Taken together, our data show that oncogenic mutant RAC1 and NRAS drive resistance to ER-stress by activating MEK/ERK signalling. These findings are important because activation of the MEK/ERK pathway is strongly associated with cancer. It has been estimated that one third of all cancers contain an upregulated MAPK signalling pathway [38] and around 16% of all cancers contain a RAS mutation [39]. This has led to the discovery and development of multiple therapeutic kinase inhibitors that target MEK/ERK signalling to prevent cancer cell proliferation and promote apoptosis [36]. Our data suggest that the use of MEK/ERK inhibitors may sensitise cancer cells to ER-stress, providing an opportunity to increase apoptosis by combining MEK/ERK inhibition with drugs that induce ER-stress, for example proteasome inhibitors or chaperone inhibitors [40]. Indeed, there is published evidence that agrees with this hypothesis: combination of RAF/MEK/ERK inhibition with HSP90 inhibition has a synergistic effect on myeloma cells [41]. The present study provides novel insight into the mechanisms of cancer cell survival under ER-stress and sheds light onto the importance of the MEK/ERK pathway in this process.

## 5 Conclusions

• RAC1 knockdown sensitises soft tissue sarcoma cells to ER-stress.
• In HT-1080 fibrosarcoma cells, N92I Rac1 controls UPR homeostasis by promoting phosphorylation of EIF2S1 and driving expression of ATF4.
• N92I Rac1 leads to constitutive activation of AKT and MEK/ERK signalling.
• In soft tissue sarcoma cell lines with constitutively active NRAS, inhibition of MEK/ERK, or knockdown of NRAS, sensitises cells to ER-stress.

## Appendix A Supplementary data

Supplementary figures Image 1
