# Supplementary material for: Oncogenic RAC1 and NRAS drive resistance to endoplasmic reticulum stress through MEK/ERK signalling
Source: Cell Signal. 2018 Apr;44:127–37. doi: 10.1016/j.cellsig.2018.01.004 (PMC6562199; doi:10.1016/j.cellsig.2018.01.004)
Supplement: Supplementary file 1 — Supplementary figures [file mmc1.pdf]

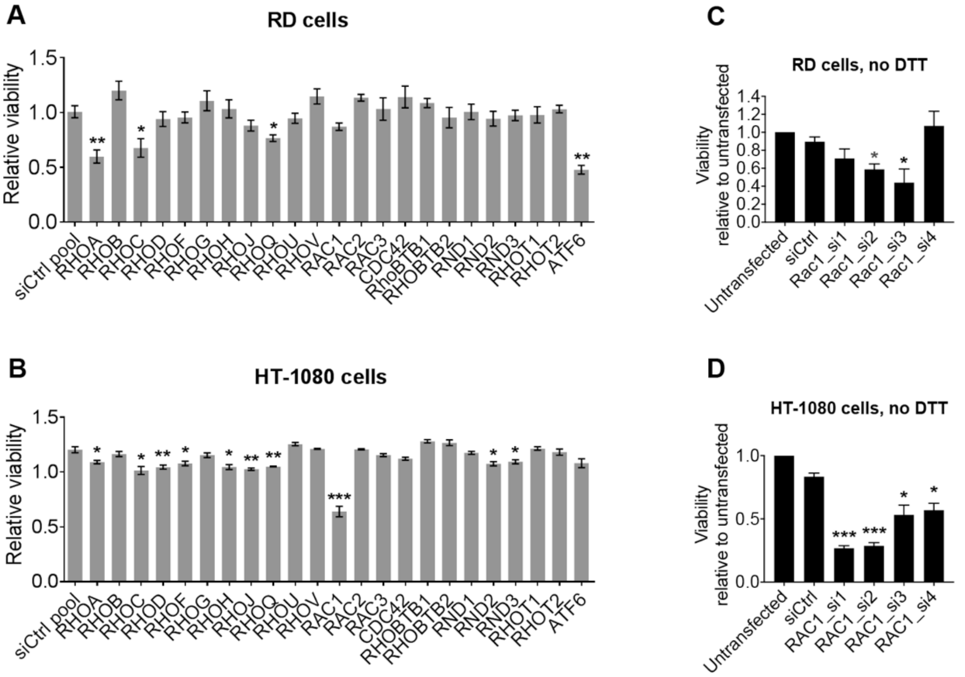

Supplementary Figure S1: Effect of siRNA on viability of HT-1080 and RD soft tissue sarcoma cell lines in the absence of ER-stressor. **A - D,** Viability of siRNA-transfected RD cells (**A and C**) and HT-1080 cells (**B and D**) relative to untransfected cells. Data are means ± S.E.M. from three independent experiments. * $p<0.05$, ** $p<0.01$, *** $p<0.001$, unpaired T-test comparing to siCtrl, n=3.

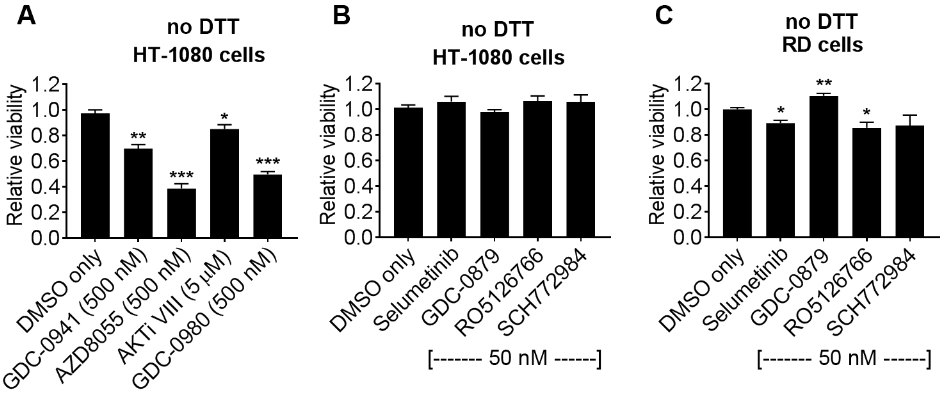

Supplementary Figure S2: Effect of PI3K/AKT/MTOR or RAF/MEK/ERK inhibitors on viability of HT-1080 or RD soft tissue sarcoma cells in the absence of ER-stressor. **A-C,** Relative viability of HT-1080 cells (A and B) or RD cells (C) treated with the indicated concentrations of inhibitor, or DMSO vehicle, for 24 hours. Data are relative to untreated cells and show mean and S.E.M. for three independent experiments. * $p<0.05$, ** $p<0.01$, *** $p<0.001$, unpaired T-test comparing to DMSO only, n=3.

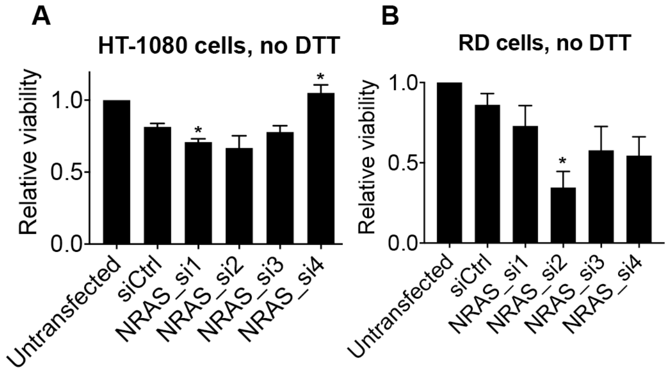

Supplementary Figure S3: Effect of NRAS siRNA on viability of HT-1080 and RD soft tissue sarcoma cell lines in the absence of ER-stressor. **A and B,** Viability of siRNA-transfected HT-1080 cells (**A**) and RD cells (**B**) relative to untransfected cells. Data are means ± S.E.M. from three independent experiments. * $p<0.05$, unpaired T-test comparing to siCtrl, n=3.
